# Supplementary material for: The Role of Science-Based Knowledge on the SARS-CoV-2 Virus in Reducing COVID-19-Induced Anxiety among Nurses
Source: Int J Environ Res Public Health. 2022 Jun 9;19(12):7070. doi: 10.3390/ijerph19127070 (PMC9222709; doi:10.3390/ijerph19127070)
Supplement: Supplementary file 1 [file ijerph-19-07070-s001.zip › ijerph-1727005-supplementary.pdf]

Supplemental Material S1:

Examples of knowledge questionnaire items:

1. You read that scientists made a breakthrough involving coronavirus antibody injections. What does this breakthrough mean?

(a) A specific treatment for COVID-19
(b) A vaccine to induce the body to produce its own antibodies
(c) Active immunity
(d) A vaccine to produce herd immunity

2. Below is a picture of people having a conversation. The grey and blue dots represent the COVID-19 virus spread. If Yossi has the virus, where do you think the virus would be?

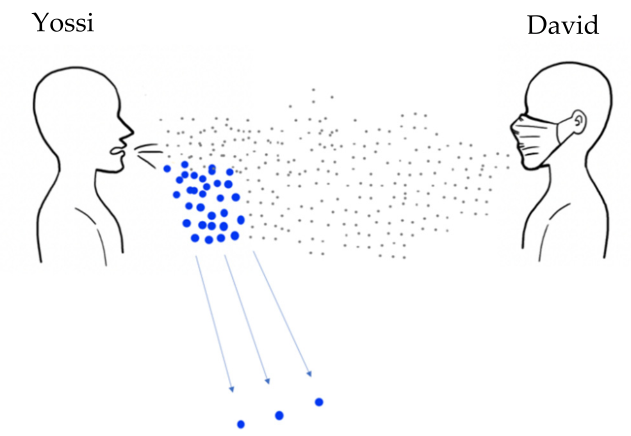

(a) Blue dots
(b) Gray dots

3. Which of the following can protect you from being infected by COVID-19?

(a) Disinfecting skin with bleach
(b) Drinking alcohol
(c) Drinking hot lemon juice
(d) Exposure to direct sunlight
(e) Washing hands with running water and soap
(f) Taking prescribed antibiotics
(g) Taking non-steroidal anti-inflammatory drugs (NSAIDs) such as Ibuprofen
(h) Smoking cigarettes
(i) Maintaining a physical distance of about six feet from another person

4. Which of the following behaviors have the highest probability of getting you infected?

(a) Pressing the elevator button without gloves
(b) Physical activity with a friend who recovered from COVID-19
(c) Staying in the same room with a person who is infected, while keeping a distance and wearing a face mask
(d) Shaking hands with a person who is infected with COVID-19 while both of you are wearing face masks.

Supplemental Material S2:

Table S1. Participating nurses' employment characteristics (*N* = 162).

Variables	Nurses
Community clinics or outpatient clinics	56 (35%)
Hospital: internal medicine or geriatric departments	28 (17%)
Hospital: neonatal care, delivery, and pediatric wards	24 (15%)
Hospital: intensive care units, emergency departments	23 (14%)
Hospital: operating rooms and preoperative care	15 (9%)
Hospital: psychiatric, dermatology, and other wards (e.g., Ear, Nose, and Throat, Dialysis, Hematology, Rehabilitation)	12 (7%)
COVID-19 wards	4 (3%)

Numbers represent n (%).
